# Invasive *Cronobacter* species infection in infants and children admitted to a rural Kenyan hospital with a high prevalence of malnutrition

**DOI:** 10.1080/20469047.2018.1446485

**Published:** 2018-03-13

**Authors:** Joe D. Piper, Salim Mwarumba, Moses Ngari, Benedict Mvera, Susan Morpeth, James A. Berkley

**Affiliations:** a Child Health, Blizard Institute, Queen Mary University of London, London, UK; b Clinical Research, KEMRI-Wellcome Trust Research Programme CGMRC, Kilifi, Kenya; c Coordination Centre, The Childhood Acute Illness and Nutrition Network, Nairobi, Kenya; d Middlemore Hospital, Auckland, New Zealand; e Centre for Tropical Medicine and Global Health, University of Oxford, Oxford, UK

**Keywords:** Malnutrition, infectious diseases, *Cronobacter*, *Enterobacter*, child, low- and middle-income countries, ready-to-use therapeutic food, UNICEF

## Abstract

For children with acute malnutrition, ready-to-use therapeutic foods (RUTF) are lifesaving treatments. In 2012, detailed testing detected Enterobacteriaceae including *Cronobacter* species at low levels in RUTF from all UNICEF-approved producers. *Cronobacter* in milk feeds has previously been associated with severe neonatal infections. Thus, given the susceptibility of severely malnourished children to invasive bacterial infections, concerns arose about the potential for *Cronobacter* infections from RUTF. This led to widespread production and supply problems in emergency feeding programmes. The KEMRI/Wellcome Trust Research Programme has conducted systematic surveillance for invasive bacterial infections among children admitted to Kilifi County Hospital, Kenya since 1998. 65,426 paediatric blood and cerebrospinal fluid cultures from 52,733 admissions resulted in 3953 with growth of a pathogenic organism. From the 60 *Enterobacter* and *Cronobacter* isolates, possible *Cronobacter* species were initially selected from their original API-20E biochemical profile, which was repeated and then confirmed using ID-32E. Only two isolates were consistent with *Cronobacter* species, neither case had received RUTF. Serious infection due to *Cronobacter* species does not have a significant burden in this population. This has important implications for the continued supply, manufacture and monitoring of emergency feeds for malnourished children.

## Introduction

For children with acute malnutrition, ready-to-use therapeutic and supplementary foods (RUTF and RUSF) are life-saving treatments. They are typically formulated from peanut paste, milk powder, oils, sugars and micronutrients, and have a low moisture content enabling a long shelf-life in tropical environments and are intended for direct consumption from the packet [1].

*Cronobacter* are Gram-negative opportunistic pathogens from the family Enterobacteriaceae [2,3]. They were previously classified as *Enterobacter sakazakii*, and then re-classified to *Cronobacter* species, including *Cronobacter sakazakii* and other *Cronobacter* species [4]. *Cronobacter* species have been isolated from environmental sources such as dust, households and food factories [5]. *Cronobacter* species can survive desiccation and may be detected despite apparent sterility for Salmonella species [6]. *Cronobacter* has also been detected in a wide range of food products, including other types of infant feeds [1,5,7].

The *Cronobacter* genus has been linked to a wide variety of human infections, including devastating outbreaks of meningitis, predominantly in neonates with case fatality ratios of up to 40% [3]. Several neonatal hospital outbreaks have been traced to contaminated formula feeds, particularly among very low birth weight and premature infants [1,4]. *Cronobacter* infections have been reported in healthy, term infants up to 2 months of age, but infection is extremely unusual in infants not fed on powdered infant formula or breast milk fortifier [3]. Infection is also highly dependent on the specific *Cronobacter* species and subtype [3]. In Holland, assuming formula feeding rates of 27% and evidence of exposure in up to 40% of formula fed infants, *Cronobacter* species were estimated to contribute only 0.5–0.7% of the meningitis burden to the Dutch population in terms of Disability Adjusted Life Years [8].

UNICEF alone supplies over 2.6 million children with RUTF or RUSF annually [9]. Given the increased susceptibility to invasive bacterial infections of severely malnourished children, concerns developed around the health implications of *Cronobacter* detected in these foods. In 2009, the most relevant criteria considered to be potentially applicable to RUTF and RUSF manufacture was the ‘Code of Hygienic Practice for Powdered Formulae for Infants and Young Children CAC/RCP 66–2008’ [10]. Although patients were older than 6 months, it was considered appropriate because of malnourished children’s susceptibility to infections. Hence, UNICEF began to apply this code to the suppliers of RUTF and RUSF [11]. The codex required the absence of any *Cronobacter* species in 10 g of powdered formula within RUTF [12]; a very strict criterion, given its widespread presence within the environment [5,11]. However, there is no validated ‘microbial kill’ step for lipid-based ready-to-use food (RUF), hence their microbiological safety is completely dependent on the microbial content of the ingredients used and the conditions in which they are prepared [1].

In 2012, Enterobacteriaceae including *Cronobacter* species were detected at low levels in RUTF from all UNICEF-approved producers [6,13] causing concern amongst UNICEF, World Food Programme and other relief agencies. For example, all the contaminated RUTF with >10 colony forming units (cfu)/gram of Enterobacteriaceae also tested positive for *Cronobacter* species [6]. Ready-to-use foods were quarantined and the RUTF obtained from local suppliers in Africa halved in 2013, with the remainder brought from Europe [9]. This led to problems for local manufacturers and supply problems in emergency feeding programmes [11]. Some stocks were also quarantined for years [1].

In 2013, the acceptable limit of *Cronobacter* species and its family Enterobacteriaceae was changed again from ‘absence’ [6] to less than ‘10 cfu of Enterobacteriaceae in 1 g’ [6,11], a limit with which producers were able to comply. In 2016, a WHO report highlighted the ongoing lack of knowledge regarding the burden of invasive *Cronobacter* disease in low- and middle-income countries (LMIC) [1], possibly because of a lack of culture-based diagnostics [1,3,14]. This knowledge gap specifically included malnourished children, receiving lipid-based RUFs [1].

The evidence for the presence of invasive infection owing to the related genus *Enterobacter* spp. among malnourished children in Africa, cited by FAO/WHO [1] was from a study conducted at Kilifi County Hospital in Kenya between 1998 and 2002, prior to the introduction of RUFs [14]. Advantage was taken of the long-term continuation of this study, providing systematic surveillance of invasive bacterial disease in sick and malnourished children, to further examine invasive disease due to *Cronobacter* and *Enterobacter* spp [14]. Now, extended surveillance over 1998–2013, including 52,081 blood and 13,345 CSF cultures from children, including those with malnutrition is reported.

## Subjects and methods

The KEMRI/Wellcome Trust Research Programme has conducted a continuous systematic surveillance for invasive bacterial infections amongst infants and children since mid-1998 [14]. Routinely, every paediatric admission or re-admission to Kilifi County Hospital (KCH) in Kenya, other than for elective surgery or observation following minor trauma, has a blood culture performed on admission, and again if there is deterioration during their hospital stay or they are subsequently re-admitted. This surveillance also encompasses children in long-term follow-up during and after treatment for severe acute malnutrition (SAM) in clinical trials.

### Laboratory procedures

An automated culture system (BACTEC, Becton Dickinson, NJ, USA) was used to incubate blood cultures and standard microbiological techniques, including API20E V4.1 profile (Bio-Mérieux, France) were used to identify the micro-organisms. Laboratory procedures are controlled internally, and externally by the UK National External Quality Assessment Service (UK NEQAS). All potentially pathogenic isolates were sub-cultured, stored at −80 °C and prospectively entered into a database linked to the clinical details of that admission.

Possible *Enterobacter* and *Cronobacter* isolates from all isolates archived between 1998 and 2013 were initially selected from the database if they had been recorded as having a potentially compatible API20E V4.1 profile (Bio-Mérieux, France). A positive control of *Cronobacter sakazakii* was obtained from an external quality assurance programme at the National Institute of Communicable Diseases (NICD) in Johannesburg, South Africa and used for quality control at each stage of testing.

After retrieval, API20E was repeated and confirmed using ID32E V3.0 biochemical profiles, as recommended in the US Food and Drug Administration Bacteriological Analytical Manual (https://www.fda.gov/Food/FoodScienceResearch/LaboratoryMethods/ucm289378.htm) where PCR is not possible. The detection sensitivity for *Cronobacter* species for the tests used has been estimated to be 90% for API20E V4.1 and 88.9% for ID32E V3.0, whilst later versions of these assays (5.0 and 4.0, respectively) which report ‘*Cronobacter* spp.’, rather than ‘*E. sakazakii*’ are reported to have lower sensitivity (15).

The selected isolates were also sub-cultured both on Tryptone Soy Agar (TSA) and *Enterobacter sakazakii* Isolation Agar (ESIA) for further characterisation, but this was not used for isolate selection. Finally, 16S rRNA gene sequencing was performed on one clinical isolate to confirm its identity, together with the positive control [5].

## Results

From 1998 to 2013, 65,426 paediatric blood and CSF cultures were processed from 52,733 admissions (Tables 1 and 2). Of these, neonates (all children less than 28 days old) comprised 17% of admissions and children with SAM represented 16% of admissions. SAM was defined using the WHO classification as either the weight for height < −3 Z-scores, or a mid-upper arm circumference < 11.5 cm, depending on age-group (see tables) or nutritional oedema. This included 8526 severely malnourished children providing 8801 cultures, of which 4319 cultures were after the introduction of RUF in 2007.

**Table 1.** Number of *Enterobacter* and *Cronobacter* species isolated from blood cultures 1998–2013 from paediatric admissions to Kilifi County Hospital, divided by demographic, nutritional status and introduction of RUTF.

	Neonates (<28 days)	Infants (29 days to 6 months) Non-SAM	Infants (29 days to 6 months) with SAM	Children (6 months to 5 years) Non-SAM	Children (6 months to 5 years) with SAM	Children >5 years Non-SAM	Children >5 years with SAM
			WHZ<-3 or nutritional oedema		WHZ<-3 or MUAC <11.5 or nutritional oedema		
							BMI Z score <-3 or nutritional oedema
	*N* = 8810	*N* = 5554	*N* = 798	*N* = 22,997	*N* = 6382	*N* = 6846	*N* = 1346
	N BC = 2961	N BC = 2500	N BC = 309	N BC = 10,782	N BC = 3111	N BC = 2455	N BC = 536
1998–2006	N POS = 188	N POS = 129	N POS = 23	N POS = 363	N POS = 239	N POS = 163	N POS = 32
(pre-RUTF)	N POS ENT = 11	N POS ENT = 1	N POS ENT = 0	N POS ENT = 6	N POS ENT = 1	N POS ENT = 2	N POS ENT = 0
	**N POS CRO** = **1**	N POS CRO = 0	N POS CRO = 0	N POS CRO = 0	N POS CRO = 0	N POS CRO = 0	N POS CRO = 0
	N BC = 5363	N BC = 2791	N BC = 390	N BC = 10,661	N BC = 2912	N BC = 3461	N BC = 539
2007–2013	N POS = 282	N POS = 352	N POS = 52	N POS = 834	N POS = 330	N POS = 213	N POS=62
(RUTF)	N POS ENT = 17	N POS ENT = 1	N POS ENT = 0	N POS ENT = 5	N POS ENT = 2	N POS ENT = 0	N POS ENT = 0
	N POS CRO = 0	N POS CRO = 0	N POS CRO = 0	N POS CRO = 0	**N POS CRO** = **1**	N POS CRO = 0	N POS CRO = 0

Notes:

The presence or absence of severe acute malnutrition (SAM or Non-SAM), weight-for-height Z-score (WHZ), mid-upper-arm circumference (MUAC), number of admissions (N), total number of blood cultures performed (NBC), number of positive blood cultures (N POS), number of *Enterobacter* identified (N POS ENT), number of *Cronobacter* identified (N POS CRO) identified in bold where present. Contaminants are excluded.

**Table 2.** Number of *Enterobacter* and *Cronobacter* CSF cultures 1998–2013 from paediatric admissions to Kilifi Hospital, divided by demographic, nutritional status and introduction of RUTF.

	Neonates (<28 days)	Infants (29 days to 6 months) Non-SAM	Infants (29 days to 6 months) with SAM	Children (6 months to 5 years) Non-SAM	Children (6 months to 5 years) with SAM	Children >5 years Non-SAM	Children >5 years with SAM
			WHZ<-3 or nutritional oedema		WHZ<-3 or MUAC <11.5 or nutritional oedema		BMI Z score <-3 or nutritional oedema
	*N* = 8810	*N* = 5554	*N* = 798	*N* = 22,997	*N* = 6382	*N* = 6846	*N* = 1346
	N C = 1180	N C = 526	N C = 74	N C = 2487	N C = 387	N C = 352	N C = 65
1998–2006	N POS = 58	N POS = 40	N POS = 9	N POS = 65	N POS = 33	N POS = 33	N POS = 9
(pre-RUTF)	N POS ENT = 1	N POS ENT = 0	N POS ENT = 0	N POS ENT = 1	N POS ENT = 0	N POS ENT = 0	N POS ENT = 0
	N POS CRO = 0	N POS CRO = 0	N POS CRO = 0	N POS CRO = 0	N POS CRO = 0	N POS CRO = 0	N POS CRO = 0
	N C = 2857	N C = 667	N C = 62	N C = 2876	N C = 318	N C = 693	N C = 98
2007–2013	N POS = 133	N POS = 57	N POS = 4	N POS = 164	N POS = 35	N POS = 44	N POS = 7
(RUTF)	N POS ENT = 9	N POS ENT = 0	N POS ENT = 0	N POS ENT = 1	N POS ENT = 0	N POS ENT = 0	N POS ENT = 0
	N POS CRO = 0	N POS CRO = 0	N POS CRO = 0	N POS CRO = 0	N POS CRO = 0	N POS CRO = 0	N POS CRO = 0

Notes:

Presence or absence of severe acute malnutrition (SAM or Non-SAM), weight-for-height Z-score (WHZ), mid-upper-arm circumference (MUAC), number of admissions (N), total number of CSF cultures performed (N C), number of positive CSF cultures (N Pos), number of *Enterobacter* identified (N POS ENT), number of *Cronobacter* identified (N POS CRO) identified in bold where present. Contaminants are excluded.

Of the 3953 positive blood and CSF cultures from the 65,426 specimens processed from 1998 to 2013, 60 isolates were identified as *Enterobacter* or *Cronobacter* species, 39 of which were in neonates. However, only two isolates, both from blood cultures, were found to be consistent with *Cronobacter* using API20E V4.1 and ID32E V3.0. These isolates were yellow on TSA and blue-green on ESIA agars. Both *Cronobacter* isolates were fully sensitive to the common antimicrobials tested (co-amoxiclav, ampicillin, cefuroxime, cefotaxime, co-trimoxazole, ciprofloxacin and gentamicin) by disk diffusion.

In addition, two further isolates consistent with *Cronobacter* on API20E profiles were identified, one from 2005 and one from 2011. These had a pale yellow colour on TSA, but had a negative ID32E profile for *Cronobacter* spp*./E. sakazakii*.

Analysis of the proportion of cultures that were positive for by age and nutritional status (Tables 1 and 2) demonstrated a low *Enterobacter* burden in children with malnutrition, with no increase in detection after the introduction of RUTF and RUSF in 2007. The clinical details of the two cases identified with *Cronobacter* infection are as follows.

In 2005, a four-day-old, fully breast-fed neonate weighing 2.2 kg was admitted with a three-day history of fever and jaundice. There was no reported exposure to infant formula or therapeutic feeding products. A diagnosis of neonatal sepsis was made and treatment with intravenous ampicillin and gentamicin was given. The infant died on the day after admission.

In 2011, a 13-month-old child was admitted with dysmorphic features suggestive of a major congenital syndrome, extreme emaciation and signs suggestive of sepsis. The child had been admitted the previous year with a life-threatening *Klebsiella pneumoniae* sepsis. The child had not received any therapeutic feeding products. Treatment with intravenous ampicillin and gentamicin was given. Again, sadly the child died.

## Discussion

Despite the largest and longest term comprehensive surveillance for invasive bacterial infections in infants and children available in sub-Saharan Africa, very few cases of invasive *Cronobacter* infection were identified. Neither case of confirmed invasive *Cronobacter* infection was related to exposure to therapeutic or supplementary foods or powdered infant formula. There was no evidence of an increase in disease due to the food-borne exposure to *Cronobacter* or *Enterobacter* species resulting from the introduction of RUTF from 2007 [1]. Of the 58 paediatric isolates that were identified as *Enterobacter* species, 39 were in neonates, which is to be expected, due to the higher neonatal susceptibility to infection [3].

It is possible that some *Cronobacter* isolates were misidentified as *Enterobacter* or other related species because the API20E and ID32E assays are not fully sensitive for the detection of *Cronobacter* species [15]. Similarly, sub-culturing isolates with a specific growth medium for pigmentation has also been reported to have a sensitivity of only 80%, and hence sub-culturing was used for further characterisation of the samples but not for isolate selection. It is possible that other samples were missed as *Cronobacter* species including the two further samples that were API20E positive but ID32E negative. Other *Enterobacter* species such as *E. hormaechei* can cause infections from ingested food and have culture profiles similar to those of *Cronobacter* [15]. Thus, the true burden of *Cronobacter* disease could be slightly higher, but the plausible number remains very low and it is very unlikely to be a burden of public health significance.

These findings help inform future decisions on the process control, production and microbiological testing for therapeutic food [1] and have important implications for the continued supply, future local manufacture and monitoring of emergency foods for malnourished children [9,11]. The data provide evidence against the hypothesis that the apparent absence of disease due to *Cronobacter* in malnourished children who are susceptible to serious infections is due to underreporting [1,3].

## Notes on contributors

***Joe D. Piper*** is a trainee in Paediatrics (ST3); an academic clinical fellow at Queen Mary, University of London; and a honorary researcher, University of Liverpool. The author’s research interests are in paediatric infectious diseases, malnutrition and growth.

***Salim Mwarumba*** is a microbiology laboratory manager. The author’s research interests are in clinical microbiology methods.

***Moses Ngariis*** a PhD Student in Biostatistics. The author’s research interests are in nutritional epidemiology, clinical trials and survival analysis.

***Benedict Mvera*** is a senior microbiology laboratory technologist. The author’ s research interests are in the clinical microbiology of invasive bacterial infections.

***Susan Morpeth*** is a consultant clinical microbiologist. The author’s research interests are in childhood pneumococcal disease, molecular diagnosis of invasive infections and hospital-acquired infections.

***James A. Berkley*** is a Professor of Paediatric Infectious Diseases. The author’s research interests are in infections and child survival in vulnerable groups, malnourished children and neonates, antimicrobial resistance and clinical trials.

## Disclosure statement

No potential conflict of interest was reported by the authors.

## Funding

This work was supported by Bill and Melinda Gates Foundation [grant number OPP1138547]; Department for International Development (GB); Wellcome Trust; Medical Research Council [grant number MR/M007367/1]; Wellcome Trust [grant number 077092].
